# Shifting to a Sustainable Dietary Pattern in Iranian Population: Current Evidence and Future Directions

**DOI:** 10.3389/fnut.2021.789692

**Published:** 2021-12-22

**Authors:** Seyyed Reza Sobhani, Nasrin Omidvar, Zahra Abdollahi, Ayoub Al Jawaldeh

**Affiliations:** ^1^Department of Nutrition, Faculty of Medicine, Mashhad University of Medical Sciences, Mashhad, Iran; ^2^Department of Community Nutrition, National Nutrition and Food Technology Research Institute (WHO Collaborating Center), Faculty of Nutrition Sciences and Food Technology, Shahid Beheshti University of Medical Sciences, Tehran, Iran; ^3^Department of Nutrition, Ministry of Health and Medical Education, Tehran, Iran; ^4^World Health Organization Regional Office for the Eastern Mediterranean, World Health Organization, Cairo, Egypt

**Keywords:** sustainable diet, dietary change, environmental footprint, nutrition, Iran

## Abstract

The need for a shift in diet toward a more sustainable one has reached an urgency in certain regions, including Iran, due to more rapid climate change and a higher level of vulnerability. This study was undertaken to identify and summarize available data on changes required in the current Iranian diet to make it more sustainable and the extent to which current policies in the country have addressed such a shift. In this study, PubMed, Scopus, and Web of science, as well as Iranian scientific search engines, including Scientific Information Database and Magiran, were systematically searched from January 1990 to July 2021. A total of 11 studies and policy analyses were included in this study. Based on the findings, moving Iranian diet toward sustainability will require increase in consumption of dairy, fruits, vegetables, cereals, poultry, and legumes and decrease in consumption of bread, rice, pasta, red meat, eggs, fats, sugars, and sweets. There has been a great deal of effort and investment on policies and strategies to decrease the amount of sugar, salt, and fat (specifically trans-fatty acids) in the Iranian diet, which makes it more sustainable healthwise. Several policies and programs have been implemented to tackle non-communicable diseases (NCDs) by reducing access to unhealthy foods, which is in line with health dimension of a sustainable diet. However, there is almost no direct address to ecological aspect of sustainable diet in the food and nutrition policy documents in the ccountry. Development of an enabling environment to a sustainable diet will require policy and actions to improve public awareness, support study to provide evidence and identify possible alternatives, and plan and implement interventions/programs to promote and facilitate healthy and sustainable diets.

## Introduction

Over the last decades, nations have experienced urbanization and consequently transformational adaptation of food systems that have resulted in substantial change in their traditional diet known as nutrition transition (1). These dietary changes have been accompanied by a higher intake of calorie and resource-intensive foods, e.g., animal products (meats and dairy), vegetable oil, and sugar (2). Based on the changes that have been happening, it is foreseen that there is 70% gap, called “food gap,” between the crop calories available in 2006 and expected calorie demand in 2050 (3). One of the options for closing this gap by 2050, as stated in the sustainable development goals (SDGs), is through shifts in food demand including shifting diets, reducing food waste, and avoiding competition from bioenergy (4). Such a shift has the potential to impact health of people and environment.

There is a growing literature on health co-benefits of sustainable diets at global, regional, national, and subnational (modeling) studies estimating the potential impact of dietary change on both the environment and health (4). According to the Food and Agriculture Organization (FAO) definition, “sustainable diets are those diets with low environmental impacts, which contribute to food and nutrition security and healthy life for present and future generations. Sustainable diets are protective and respectful of biodiversity and ecosystems, culturally acceptable, accessible, economically fair and affordable, nutritionally adequate, safe, and healthy, while optimizing natural and human resources” (5). Recent studies have shown that diets comprising reduced animal sources (specifically reduced red and processed meat) and high levels of plant-based foods including fruits and vegetables are not only associated with decrease in non-communicable diseases (NCDs) (6, 7), but can result in lower environmental footprints (8, 9).

The need for a shift toward a more sustainable diet may have more urgency in certain regions. An example is Iran, a middle-income country located in the Middle East that is mostly an arid and semi-arid region where climate change is occurring more rapidly. It is expected that the amount of precipitation in this region drop by 20% within the next century, which makes the region more vulnerable (10). In addition, over the last four decades, due to industrialization and rapid urbanization, nutrition transition and major changes in the food system in the country have occurred that put further pressure on the environment as well as health of the population (11). Therefore, considering the urgency of the problem and the fact that there is not one sustainable diet, there is a need to define the most sustainable choices based on food availability and dietary pattern in each region (12).

There are relatively limited data available on sustainability aspects of the Iranian diet. This study was undertaken to identify and summarize available data on changes required in the current Iranian diet to make it more sustainable and the extent to which current policies in the country has addressed such a shift.

## Methods

### Conceptual Framework

Sustainable diets are diets that consider how the food system influences health, environmental outcomes, and vice versa (5). Therefore, sustainable healthy diets are dietary patterns that promote all the dimensions of health and well-being of an individual; in the meantime, sustainable healthy diets have low environmental pressure and impact and are accessible, affordable, safe, equitable, and culturally acceptable (5). Sustainable healthy diets aim to achieve optimal growth and development of all the individuals and support functioning and physical, mental, and social well-being at all the life stages for present and future generations; contribute to preventing all the forms of malnutrition (i.e., undernutrition, micronutrient deficiency, overweight, and obesity); reduce the risk of diet-related NCDs and support the preservation of biodiversity and planetary health (12). In other words, sustainable healthy diets must combine different dimensions of sustainability to avoid unintended consequences, thus shifting from “current” to more “sustainable diets” that could serve as both a climate mitigation strategy and improved population health. For these reasons, shifting to a sustainable diet has been proposed as one of the core strategies to achieve SDGs (5).

**Figure 1:**
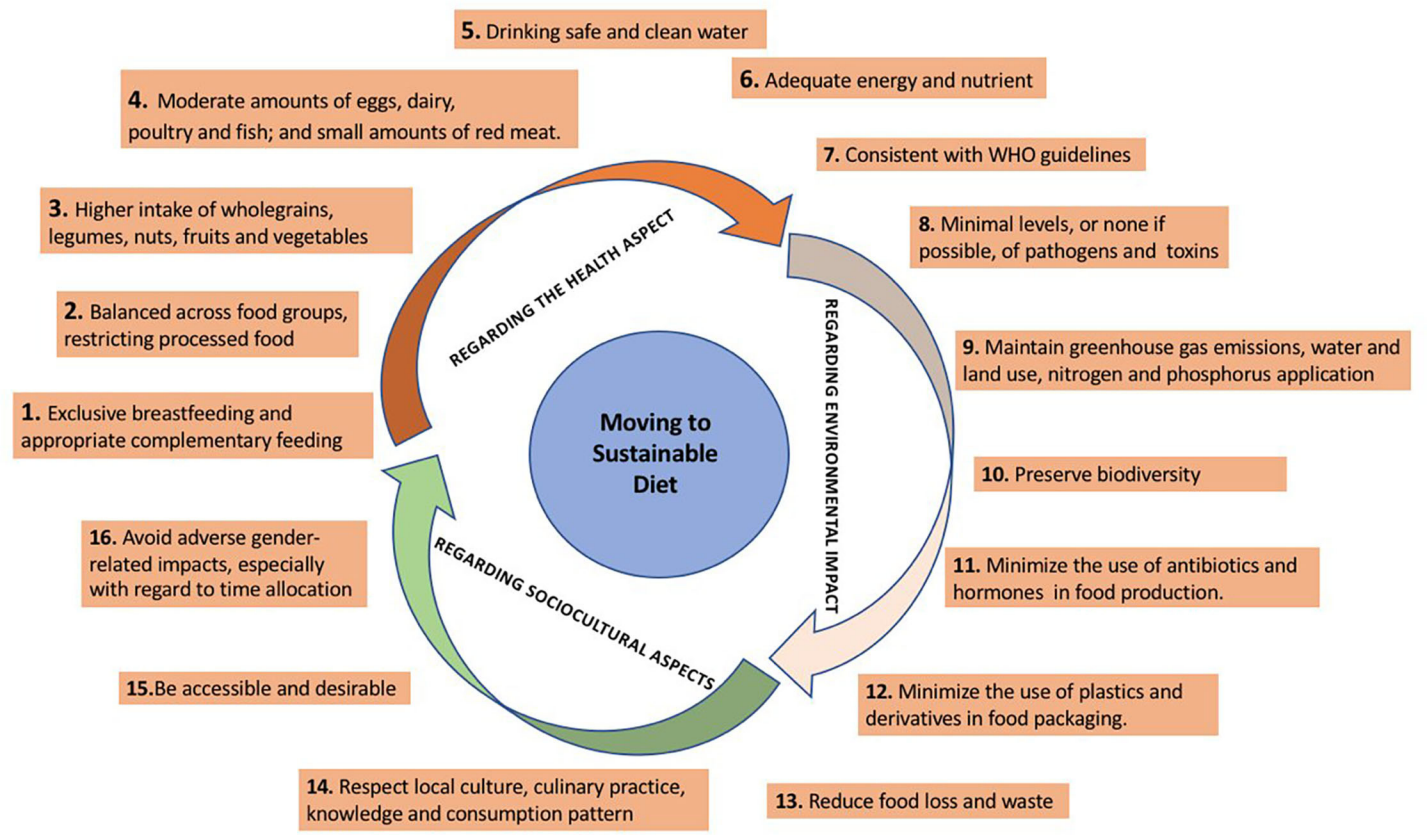
Conceptual framework of shift to a sustainable diet (12).

Despite these facts, reaching a sustainable diet is a complex process that requires taking several steps to consider nutrient recommendations as well as environmental, social/cultural, and economic sustainability. Figure 1 presents a conceptual framework adapted from “Guiding Principles for Sustainable Healthy Diets” developed by the FAO and the World Health Organization (WHO) (12) as a basis for this study. Based on this framework, in order to shift to a sustainable diet, various actions with respect to health, environmental, and sociocultural aspects of diet, including changing intake of different food groups, should be taken.

## Methodology

### Identifying the Study Question

This study aimed to answer the following questions:

- To what extent the topic of “sustainable diets” has been addressed in studies conducted in Iran?
- What is the most widely used definition of a sustainable diet in Iranian studies and which dimension of diet sustainability has been studied most?
- To what extent are diets in Iran in accordance with the principles of a sustainable diet?
- Has the issue of sustainable diet been considered/addressed in food and nutrition policies/guidelines in Iran?
- What policies and programs are needed in moving toward a sustainable diet in Iran?

### Identifying Relevant Studies

We systematically searched articles related to sustainability of diet/nutrition/food system in Iran, using PubMed, Scopus, Web of science, and two Persian scientific search engines: Scientific Information Database (SID) (www.sid.ir, accessed on 15 July 2021) and Magiran (www.magiran.com, accessed on 15 July 2021).

The literature search was adapted to the databases and included the following subject heading, terms, and keywords: (carbon footprint or water footprint or land footprint or environment or sustainability or resilient or biodiverse or ecologic or life cycle analysis or global warming or climate or greenhouse gas (GHG) or greenhouse) and (food or diet or nutrition or consumption) and (Iran). We limited the search to the following dates: 1st January 1990 to 15th July 2021 and applied no language restriction. Additional references were identified by searching the gray literature and hand searching the reference lists of the included articles.

### Study Selection

All the articles (in Farsi or English) that had information related to sustainable diet in Iran were included in this study. Studies that were related to sustainability issues other than food, nutrition, and/or diet or conducted in countries other than Iran were excluded. Screening of titles and abstracts was followed by full-text screening and data extraction.

### Charting the Data

The data extraction table was designed to record the following variables: year of publication, aims, food item(s)/diet, sustainability dimensions measured, changes suggested in the diet, and finding(s).

### Collating, Summarizing, and Reporting Results

Included studies were reviewed and findings with respect to the questions of the study were collected and summarized. In all the stages, two of the authors (SRS and NO) held regular meetings to discuss findings and reach a consensus about the management of the findings.

## Results

**Figure 2:**
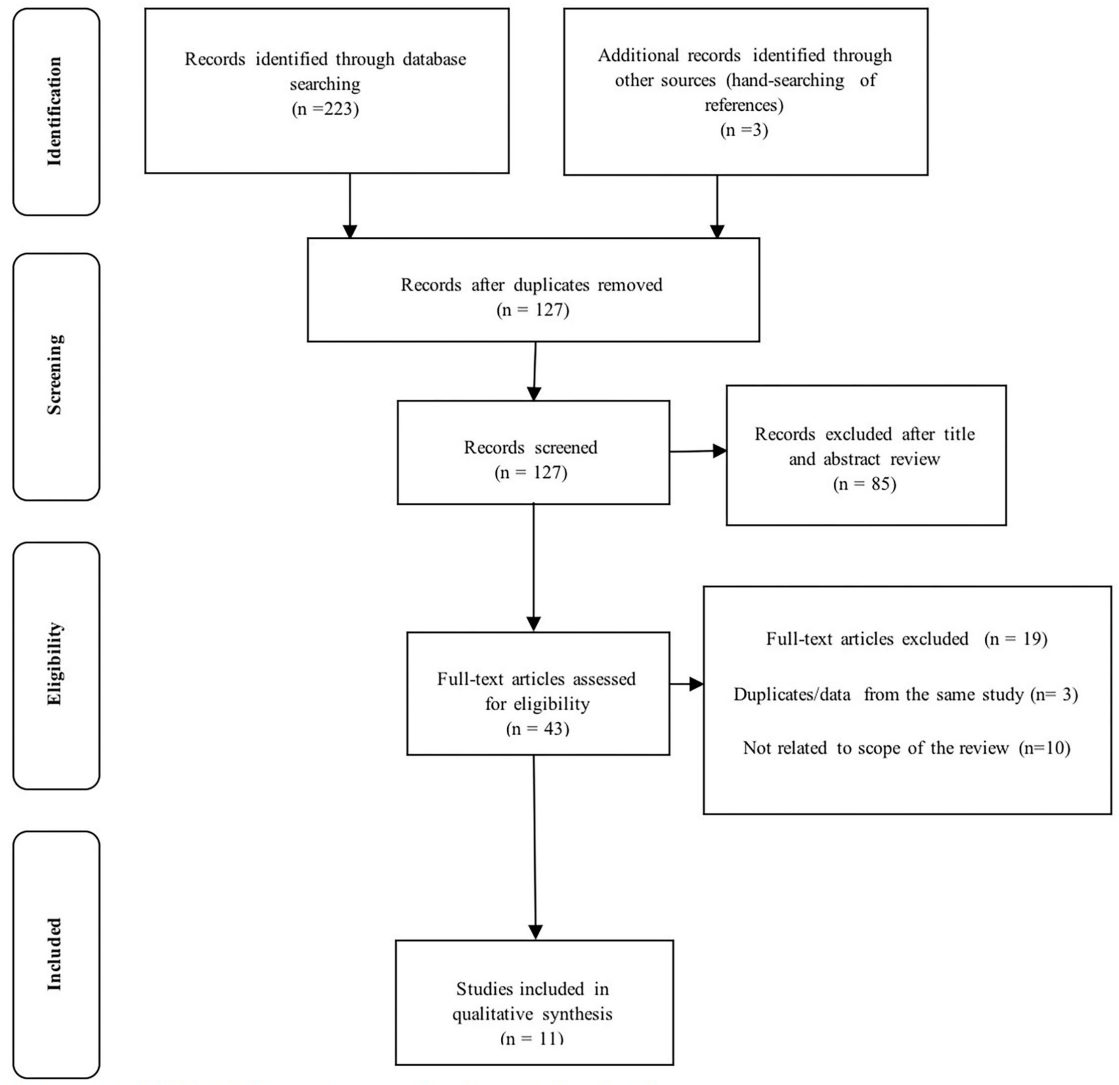
The PRISMA flow diagram for the study selection process.

As illustrated in the Preferred Reporting Items for Systematic Reviews and Meta-Analyses (PRISMA) diagram (Figure 2), 226 records were identified, of which 127 records were screened and excluded due to duplication. By reviewing the title and abstract, 42 records satisfied eligible criteria that were chosen for their full text to be read. Finally, 11 studies met the inclusion criteria and were included in this study (Table 1).

**Table 1:** Summary of characteristics of studies on sustainable diet in Iran (1990–2021).

**First author (year)**	**Aim**	**Food item(s)/diet**	**Sustainable diet dimension(s) measured**	**Suggested change(s) in diet**	**Finding(s)**
Rahmani et al. (13)	To determine the impacts of dietary changes on the Iranian economy and on the environmental load.	Total diet, based on food balance sheet	• Carbon FP • Cost • Nutritional value	– Decreasing rice, vegetables, fruit, bread and pasta – Increasing livestock and other animal products	• A shift from the current diet to alternative diets increases both the economic output and the environmental pressure. • Compared to the Mediterranean dietary pattern, shifting Iranian dietary patterns toward WHO and WCRF has a greater positive effect on economic output, but a more negative impact on the environment.
Eini-Zinab and Sobhani (14)	To compare the sustainability of traditional and local foods in Iran with Western Foods	Three traditional Iranian cusines (*Ashe Reshteh, Mirza- Ghassemi* and Tbrizi Koofteh) and three western foods (Pizza, Beef-estroganove and Pasta)	• Carbon FP • Water FP	Increase consumption of traditional and local Iranian foods/cuisines	• Food with high contribution of animal products had the highest carbon footprint. • Traditional and local Iranian foods seem to be more sustainable with low environmental effects compared to the selected western foods. • Traditional food patterns could be promoted through food and nutrition policy to achieve sustainable food and nutrition systems.
Sobhani et. al. (15)	To assess the compliance of the Iran's National Nutrition and Food Security Policy (INNFSP) with the components of the sustainable diets framework	National policy	–		• The compliance of the INNFSP with the components of a sustainable diet, without weighting importance and adequacy, was about 60.32%. The score was 60.69% when importance was weighted. • The percentage of compliance with the components of a sustainable diet was 41.79% when both importance and adequacy weighted. • In order to achieve a sustainable diet; which in addition to providing nutritional needs, has environmental, cultural, and economic sustainability; national food and nutrition policies needs to consider dimensions other than nutrition and health, as well.
Kalvani et. al. (16)	To evaluate and analyze water footprint of 14 important crops in Tehran province and to assess their water savings and losses in 2008–2014.	Apple, barley, bean, grapes, maize, onions, oranges, potatoes, rice, tomato, wheat, cherry, pear, peach	Water FP	Decrease wheat/wheat products	• Wheat and rice had the largest per capita water footprint • The consumption of wheat in Iran is high (2 times larger than global average). • It is recommended to reduce the consumption of wheat in Tehran or replace it with other crops.
Soltani et. al. (17)	To investigate the role of current diets and types of modified diets on the need for environmental resources such as water, land, and inputs, including nutrients, energy, and greenhouse gas emissions.	Total diet	• Water FP • Carbon FP • Fertilizers use • Cost • Energy • Nutritional recommendation	Decrease rice (67%), potato (54%), oil (30%), sugar (53%), red meat (50%), chicken (55%), egg (48%), fruit (6%), and Increase wheat flour (43%), legumes (78%), and vegetables (32%)	• Plant-based diet can reduce the need for blue water resources by 30%, fertilizer by 8–12%, energy by 15%, and greenhouse gas emissions by 10-14%. The cost of these diets are also 20–24% less; • Implementing and adopting sustainable plant-based diets requires cultural and educational efforts.
Sobhani et. al. (18)	To assess different scenarios to reduce water use by following healthy diet recommendations/ to suggest a healthy diet with low water FP for the Urmia population.	Total diet in Urmia based on a FFQ	• Water FP • Nutritional value • Cultural acceptance	Decrease “bread, cereal, rice, and pasta” (14%) and meats (3%) Increase dairy (14%) and fruits (6%)	A healthy diet with greater proportion of energy from fruits, and lower ratio from “bread, cereal, rice, and pasta”, and substitution of meats with beans can supply all recommended dietary allowances while reducing water use by 49%.
Mirzaie-Nodoushan et. al. (19)	To investigate the effects of diet change on reducing water consumption in Iran, while meeting its nutrition requirements.	Iranian food basket	• Water FP • Nutritional recommendations • Cultural acceptance	Decreases red meat (47%), fruits (35–44%), poultry (9–42%), vegetable oil (13–25%), sugar (26–30%), and rice (17–40%). Increase vegetables (80%), milk (78–80%), pulses (51–75%), fish (29–80%), eggs (37–79%), and wheat (15–21%)	These changes resulted in 7.9%-16.7% decrease in water footprint.
Eini-Zinab et. al. (20)	To develope a healthy, low-cost and environmental-friendly food basket for Iran, based on current consumption	Total diet	• Water FP • Carbon FP • Cost • Nutritional recommendation • Cultural acceptance	Decrease the “bread, cereal, rice, and pasta” (34%), “meat, poultry, fish, eggs, legumes, and nuts” (11%) and “fats, oils, sugars, and sweets” (24%) Increase dairy (34%), fruits (26%) and vegetable (8%) groups and cereals (38%), poultry (45%) and vegetable oil (30%) subgroups	In the sustainable diet model extracted, there was a 14% reduction in total water footprint, a 14% decrease in total carbon footprint, and a 23% decrease in the cost, as well as 7% increase in NRF of diet compared with the usual consumption.
Edalati et. al. (21)	To analyze a canteen menu of the School of Nutrition Sciences and Food Technology sustainability and to develop a sustainable lunch menu	A campus lunch menu	• Water FP • Carbon FP • Land FP • Cost • Nutritional recommendation • Cultural acceptance	Decrease red meat Increase chicken or fish and vegetables	Replacing the sustainable food menu designed for Menu 1(rice-based) could decrease carbon, total water and land footprints and costs by 10, 13, 22 and 6%, respectively, and increased the NRF profile by 8%. Replacing the sustainable menu designed for Menu 2 (vegetable or meat-based, no rice but with wheat bread) could result in 25, 23, 27 and 28% decreases in carbon, total water and land footprints and costs, respectively, and increased the NRF profile by 23%.
Noormohammadi et.al. (22)	To suggest dietary scenarios for decreasing GHG emissions	Total diet	• Carbon FP • Nutrition recommendation • Cultural acceptance	Decrease red and white meats, eggs, grains, fats and oils, and sweets Increase vegetables, fruits, legumes, nuts, and dairy.	• A healthy diet with a higher proportion of vegetables, fruits, legumes, nuts, and dairy, and a lower share of red and white meats, eggs, grains, fats and oils, and sweets can reduce carbon FP by %50.
Sobhani et. al. (23)	To evaluate the sustainability of Iranian FBDG in comparison with the usual diet and with the selected food pyramids.	Total diet	• Water FP • Carbon FP • Land FP • Cost • Nutritional recommendation • Cultural acceptance	Increase legumes and nuts	• Replacing the usual dietary intake of the Iranians with an optimal diet based on the 2016 Iranian FBDG was associated with reductions equal to 20.9 % for water footprint, 22.48 % for carbon footprint, 20.39 % for land footprint, 31.83 % for cost, as well as 7.64 % increase in NRF index.

*FP, footprint*.

### “Sustainable Diets” Definition and Measurement in Studies in Iran

The results indicated that “sustainable diet” is a topic that is being addressed by researchers in Iran in the recent years. Except for one study by Rahmani et al. in 2011, all the included studies had been conducted between 2017 and 2021 (13). Almost all the included studies were focused on the effect of dietary consumption change on the sustainability of diet (13, 14, 16–23), except one (15) that assessed the compliance of national nutrition policy with the sustainable diet framework. Of 10 studies on measuring sustainability dimensions of diet and changing it toward sustainability, 7 studies had focused on total dietary consumption (13, 17–20, 22, 23), one on a campus lunch menu (21), one on 14 important crops in Iran (16), and one compared the sustainability of traditional Iranian dishes with western dishes (14).

With respect to different dimensions of the sustainable diet, all the 10 studies had considered one or more of the environmental dimensions, specifically water footprint (14, 16–20, 23) and carbon footprint (13, 14, 17, 20–23). However, energy (17) and land footprint (21, 23) each were measured only in one and two studies, respectively. In addition, seven studies evaluated fulfillment of nutritional recommendations (17–23) and cost and cultural acceptance of diet were considered in five (13, 17, 20, 21, 23) and six (18–23) studies, respectively.

### Dietary Changes Required to Move Toward a Sustainable Diet in Iran

The main approach used to identify what changes are needed to make the diet more sustainable was through comparing available data on Iranian food consumption and/or acquisition including food balance sheet data (13), household food expenditure (19, 20, 23), and food intake data (17, 18, 21, 22) with a designed and/or recommended sustainable diet. In order to design a sustainable diet, an optimizing method, i.e., linear programming (18, 19, 22), goal programming (20, 21, 23), or a recommended reference diet (13, 17) was used. The included studies based on their main purpose can be classified into three groups proposing a sustainable diet: (1) to reduce carbon footprint (13, 22); (2) to reduce water footprint (16, 18, 19); and (3) to fulfill nutritional, environmental, and economical criteria (17, 20, 21, 23). Some details of the findings are as follows:

Noormohammadi et al., using linear programming, showed that compared to the usual intake (in Urmia city, in north-west Iran), a healthy diet with a higher proportion of vegetables (20%), fruits (25%), legumes (7%), nuts (45%), and dairy (115%) and a lower share of red and white meats (92%), eggs (70%), grains (42%), fats and oils, and sweets (12%) can lead to about 50% decrease in carbon footprint. The recommended diet was nutritionally adequate, although its cost was not measured (22). Rahman et al. (13), using food balance sheet data of Iran, showed that a shift from the current diet to alternative diets, based on the WHO, the World Cancer Research Fund (WCRF), or the Mediterranean diet recommendations, will require to decrease the amount of rice, vegetables, fruits, bread, and pasta and increase comsumption of animal products. They showed that such changes will result in positive effects on economic output, while it has a negative environmental impact due to increasing carbon footprint (13). On the other hand, studies that considered carbon footprint in addition to other dimensions of the sustainable diet have shown that diets with higher consumption of vegetables, legumes, and cereals and lower contribution of red meat, rice, sugar, and egg can reduce greenhouse gas emissions by 10–14% as well as cost (20–28%) compared to the usual diet (14, 17, 20, 21).

With regard to minimizing water footprint of diet, a study by Sobhani et al., using linear programming, showed that including a greater proportion of energy from fruits (6%) and dairy (14%) and decreasing the energy share of bread, cereal, rice, and pasta (14%) as well as meat, fish, poultry, and eggs (3%) can supply all the recommended dietary allowances while reducing water use by 49% (18). Although cultural acceptance of dietary change was considered through minimizing the distance between an optimal diet with the usual diet, other environmental indicators (e.g., carbon or land footprint) or costs were not calculated. Similarly, in a study by Mirzaie-Nodoushan et al. (19), the minimized total water footprint scenario of 7.9 to 16.7% can be resulted by increasing intakes of vegetables (80%), milk (78–80%), pulses (51–75%), fish (29–80%), eggs (37–79%), and wheat (15–21%) and decreasing red meat (47%), fruits (35–44%), poultry (9–42%), vegetable oil (13–25%), sugar (26–30%), and rice (17–40%). Another study that included carbon footprint and cost in addition to water footprint, nutrition recommendation, and cultural acceptance reported a 14% reduction in total water footprint (20). Soltani et al. showed that 30% reduction in blue water footprint, i.e., surface and groundwater during the production of a product and through its supply chain can occur by following a plant-based diet (with lower rice, potato, oil, sugar, red meat, chicken, egg, and higher amount of wheat flour, legumes, and vegetables consumption compared to the usual diet) (17).

On the other hand, Edalati et al., in an attempt to design a sustainable lunch menu for a university campus in Tehran, showed that compared with the usual menu, including a higher amount of bread and vegetables while decreasing rice and red meat in the menu, it leads to 23% decrease in water footprint (21). Further, it has been shown that a higher share of bread and vegetables in traditional and local Iranian foods lead to a lower water footprint compared with western foods (14). While most studies have recommended reducing rice consumption (18, 19), Kalvani et al., analysis of water footprint of 14 important crops in Tehran province, recommended reducing the consumption of wheat, which is mainly consumed as white flat bread and suggested replacing it with other crops in order to reduce water FP (16).

A total of four studies simultaneously considered dimensions of sustainable diet including nutritional, environmental, cost, and cultural acceptability in their analysis (17, 20, 21, 23). Despite the differences in findings, almost all emphasized on higher intake of dairy (17, 20), vegetables (17, 20, 21), cereals (17, 20, 21), fruits (20), and legumes (17, 21, 23) and in the meantime, lower intake of meat (17, 20, 21), rice (17, 20, 21), sugar (17, 20, 21), fats (17, 20, 21), and egg (17, 20, 21) in order to have a sustainable diet. With respect to poultry consumption, there was inconsistency in the results; one study recommended higher consumption (20), while the other study emphasized reducing it (17) in order to have a more sustainable diet in Iran.

In a study by Sobhani et al., aiming at developing a healthy, low-cost, and environmentally-friendly food basket, an optimal food basket compared with the usual consumption, resulted in 14% reduction in both the total water footprint as well as total carbon footprint, 23% decrease in the cost, and 7% increase in nutrient rich foods (NRF) of diet (20). Soltani et al. suggested that a plant-based diet can reduce the need for blue water resources by 30%, fertilizer by 8–12%, energy by 15%, and greenhouse gas emissions by 10–14%. Also, the diet cost 20–24% less (17). Cultural acceptance was not considered in the latter study and this may explain the difference between its findings with those of Sobhani et al. (20). Also, the sustainable lunch menu proposed by Edalati et al. included more dishes based on chicken or fish with vegetables and fewer red meat and rice-based dishes. They showed that this change can result in decreasing carbon, total water, and land footprints as well as costs of the lunch menu by 25, 23, 27, and 28%, respectively, and 23% increase in its NRF profile (21).

Finally, evaluating the sustainability of the first (2005) (24) and second (2016) (25) versions of national Food-Based Dietary Guidelines (FBDGs) in Iran in comparison with the usual diet showed that while both the FBDGs have lower cost, water and carbon footprint, and higher nutritional value, the difference was statistically significant only for the more recent version. The second national FBDG emphasizes legumes and nuts by defining them as a separate food group from the meat group. Replacing the usual dietary intake of the Iranians with the optimal diet based on the last Iranian FBDG (2016) was associated with reductions equal to 20.9% for water footprint, 22.48% for carbon footprint, 20.39% for land footprint, 31.83% for cost, and 7.64% increase in NRF index (23).

### Considering Sustainable Diet in Food and Nutrition Policies in Iran

No direct address to “sustainable diet” in the food and nutrition policy documents in Iran was found. However, a recent analysis on the National Food and Nutrition Security Policy compliance in Iran with the components of a sustainable diet reported a score of 60% (15). Through the mentioned policy document, some of the strategies needed to achieve a sustainable diet have been recommended including use of effective economic tools to promote healthy nutrition (e.g., sin tax, subsidies, and loans), obligatory and optional nutrient fortification of the main and complementary foods, healthy formulation of produced foods, promoting home vegetable gardens in rural areas, and distribution and supply of vegetables, fruits, and legumes in remote areas of the country through rural cooperatives to increase access of community to micronutrient resources and finally redirecting food subsidies to increase consumption of vegetables, fruits, meat, milk, and dairy products (26). Similar recommendation has been also provided by the National Action Plan for Prevention and Control of NCDs in Iran aiming at 30% reduction in sodium/salt intake and zero amount of trans-fatty acids in oils and food products by 2025 (27). However, cost and ecological aspects of diet were not taken into account in both the documents.

## Discussion

“Sustainable diets” are not mentioned directly and clearly in the SDGs; however, according to the post-2020 global biodiversity framework, in achieving the vision of “living in harmony with nature,” shifting toward a sustainable diet is one of the key actions required (28). This study is an attempt undertaken to summarize current literature on the sustainability of the Iranian diet in order to inform decision-makers on changes required to dietary guidelines and policies to be both nutritionally adequate and environmentally sustainable and to identify potential trade-offs.

In Iran, given the fact that there is low intake of vegetables, fruits, and dairy (29), a large share of food in the household budget (about 24%) (30) and an increasing trend of water shortage (31), CO_2_ emission (32), and warmer climate (33) during the recent years, moving toward a sustainable diet, is inevitable. The findings of this study showed that an increase in dairy, fruits, vegetables, cereals, poultry, and legumes consumption and a decrease in intake of bread, rice and pasta, red meat, eggs, fats, sugar, and sweets are the main changes being suggested in the Iranian diet in order to move it toward sustainablility. These findings are in line with the EAT-Lancet recommendation that emphasizes a diet rich in plant-based foods with fewer animal sources in order to both improve the health and benefit of the environment (4). The specific recommendations identified with respect to the required changes in the Iranian diet are as follows:

First, the need for consuming higher amounts of varied fruits and vegetables as a step toward a sustainable diet was reconfirmed in this study. Sustainable diets are characterized by varied and high amounts of vegetables and fruits (34), which make them more nutritious and environmentally friendly, with a lower risk of chronic diseases compared to other dietary patterns (35). Vieux et al. investigating changes needed to improve sustainability of the diet across Europe and concluded that this can be achieved by substituting food items from the sugar/fat/alcohol group with fruits, vegetables, and starches in “the diet” (36). It is worth noting that consumption of fruit and vegetables in Iran has been consistently reported to be lower than minimum recommended amount of 400 g per capita per day by the WHO (29, 37). Lack of access to fruits and vegetables in all the regions and in all the seasons of the year, high price of fruits in particular, and the high loss and waste rate of these products are the identified reasons behind insufficient intake of these food groups in Iran (38).

Second, reducing red meat consumption and substituting it with legumes (plant sources of proteins) or poultry in the Iranian diet is another recommendation that has been raised by the studies on sustainable diet in Iran. Since 1960s, consumption of animal-based foods has increased throughout the world due to rising global average income, higher standards of living, and increased efficiency of production (35). Similarly, the share of meat in the Iranian food basket has increased over the past 30 years; although in the recent years, due to the economic crises of country, the increase rate has slowed down (29). Bahn et al. had previously proposed that reducing red meat consumption and simultaneously increasing consumption of vegetables/beans in Middle East and North Africa (MENA) countries can result in positive environmental effects (39). Considering higher water and carbon footprint, energy input, fertilizer, and pesticide use with an animal-based diet compared with a plant-based diet (35), low-to-moderate consumption of seafood and poultry and zero to low consumption of red meat and processed meat are recommended in order to have a sustainable diet (4). With lower water Footprint (FP) and price of poultry compared to red meat, it seems poultry is a more realistic choice (40, 41). Fish is anoter alternative, which is not much emphasized in Iranian population due to its higher price and low desirability in the majority of Iranians (it is highly desirable in North and South of Iran). Average consumption of fish in the Iranian diet (8 g/day) (29) is way below the recommended amount of 30 g/day (42).

Another suitable alternative to red meat is legumes. Legumes are inexpensive and sustainable sources of protein with low carbon and water FP that are part of the traditional diets in most cultures including Iran (43). Based on a study by Roos et al., reducing daily per capita intake of meat from 110 to 55 g and replacing it with 50 g of beans is associated with a 20% reduction in environmental footprint (44). Besides, legumes production positively affects soil quality, biodiversity, and enriches the soil through nitrogen fixation (45). The role of legumes in achieving a sustainable diet is so important that the FAO designated 2016 as the year of pulses as a subgroup of legumes and introduced it as food crops that can play a major role in addressing future global food security and environmental challenges as well as contributing to healthy diets (46). For these reasons, in the second version of FBDG in Iran (2016), legumes and nuts have been defined as a separate food group from the meat group. Comparing the sustainability dimensions of the first and second FBDGs of the country showed that the recent version can result in a diet with lower cost, water, and carbon footprint, provides higher nutritional value, and can be more in line with a sustainable diet compared to the previous version (2005) (23).

Third, replacing refined carbohydrates (e.g., white bread, polished/white rice) with whole-grain products is another required step identified to move toward a sustainable diet in Iran. White rice and white flat bread are the main staple foods in the Iranian diet, the share of the latter being higher among those with lower income (47). Iran is the 13th biggest white rice consumer worldwide (47) with an annual consumption of 36.6 kg per capita, which is 7 times more than the European Union (5.3 kg per capita) (48). Higher white rice and bread intake are associated with an increased risk of type 2 diabetes (49, 50). Besides, wheat and rice have the largest per capita water FP in the country (16). It should be noted that water FP varies for each crop depending on irrigation and on average rice cultivation has the least water efficiency (51). Studies on the sustainable diet in China also showed that a decrease in rice consumption is required due to its role as the largest contributor to reductions in GHG emissions (52). Promotion of less refined and polished rice or wheat will require policy change at the production level as well as consumer education.

The fourth recommendation identified through this study in order to have a more sustainable diet is decreasing sugar and fats. Consumption of oils and sugar by Iranian is 20 and 38% of daily energy intake, respectively, which is higher than the recommended amounts (53). Since 1960s, due to the improvement of vegetable oil production technology and increased supply, its price has decreased worldwide (54). Also, the increased supply of processed foods and sweetened beverages has resulted in higher intake of sugars (55). Despite low environmental impact of sugar and fat consumption, considering their negative impact on health and prevention of NCDs (37), the need for reducing their intake to 31 g/day for added sugar and 40 g/day for added fats has been emphasized (4).

The final recommendation is the only one that seems differerent with the EAT-Lancet recommendation of decreasing dairy products; however, due to very low consumption of dairy products in Iran (168 g/day) (27), there is a need to increase the consumption of this food group in order to comply with the recommendation of 250 g/day (29). In fact, the average consumption level of dairy products in Iran is less than half the recommended level and has had a decreasing trend due to a drastic rise in the price over the last decade as a result of inflation and poor governmental support policies (56). Similarly, Donati et al. have recommended increasing dairy as part of the changes required to achieve a more sustainable diet in adolescents in Italy (57).

Based on this study, there is no clear and direct reference to sustainable diet in the related documents in Iran and there is no consistent approach with respect to the policies and strategies to move toward a sustainable food and nutrition system in policy documents in Iran. In one hand, the food production policies of country have been more focused on increasing agricultural production aiming at self-sufficiency and sustainable agriculture with less attention to the quality and sustainability of the food consumed, i.e., sustainable diet (58). On the other hand, due to the high prevalence of NCDs, the health sector has properly focused on health aspect of a sustainable diet through investment on policies and strategies to decrease the amount of sugar, salt, and fat (specifically trans-fatty acids) in the Iranian diet. The National Action Plan for Prevention and Control of NCDs in Iran has strongly focused on reducing sodium/salt intake as well as saturated and trans-fatty acids in oils by 2025 (27). In line with these policies, several regulatory policies/programs have been implemented to tackle NCDs, e.g., setting a mandatory upper limit of salt in bread (59, 60), food reformulation initiatives for reducing the amount of sugar, salt, and fat, specifically the industrially-produced trans-fatty acids in industrial food products (61, 62), sin tax policy on increasing the price of sugar-sweetened beverages (SSB) (63), traffic light nutrition labeling for more than 80% of the manufactured food products (64), and educational interventions/campaigns to improve awareness and food choices of consumers (65). In addition, the national FBDG, as one of the main tools to raise awareness with respect to appropriate and healthy diet, has been in place since 2005 (24). However, within all these policies, there is a gap in adressing ecological aspects of a sustainable diet (e.g., water, land, and carbon footprints) with a specific reference to context of Iran. Recently, there have been attempts to evaluate all the dimensions of sustainablility of Iran FBDG (23) to pave the ground for a sustainable FBDG in the close future.

One other aspect that is almost ignored in study with respect to sustainable diet in Iran is measurement and interventions with respect to food loss and waste. There are different estimations of the amount of waste and loss of agricultural products in Iran, ranging from 18.5 up to about 35% (66). A report by the Food Producers Cooperatives of Iran indicates that out of about 130 million tons of foods being produces in Iran, 25 million tons are lost (67). According to the study conducted by Fami et al. in Tehran, every consumer wastes about 27 kg of edible food annually (66). A total of seven food items identified with the highest amount of waste at household level are bread, cooked rice, cooked pasta, fresh fruits, fresh vegetables, salads, milk, and dairy products (66). Developement of proper infrastructure and skills to decrease the loss at the production and distribution levels as well as improving knowledge and skills on planning, preparation, and storage, and raising awareness about values and consequences of avoiding food waste at the procurement and consumption levels (66) can lead to decrease food loss and food waste.

Table 2 presents the current policies across the food system that support move toward a sustainable diet in Iran, based on the available recommendations (68) as well as gaps and recommended policies to improve current efforts.

**Table 2:** Regional recommended strategies for a sustainable food diet, current status of related policies in Iran, and recommended actions.

**“Game changing” food systems actions (68)**	**Recommended priority actions 2020–2030**	**Existing policies in Iran**	**Gap/recommended policies for Iran**
Fiscal policies for healthy and sustainable diets.	• Implement a tax on sugar-sweetened beverages and use other taxes and subsidies to promote healthy diets • Review food subsidy programs and progressively eliminate subsidies for all types of fats/oils and sugar.	• 2015: A maximum tax of 10% was imposed to unhealthy food (63). • The Iranian budget law for the fiscal year 2013–2014 obligated the government to taxation of soft drink at rate of 15 and 20% for locally produced and imported goods, respectively (63). • 2010: Subsidy on sugar and vegetable oil were reduced (69). • 2014: Eliminating subsidies for milk. (70)	Elimination of subsidies on vegetable oil and sugar and instead shifting subsidies to healthier foodstuffs (i.e, fruits, vegetables and dairy) (71).
Public food procurement and service policies for a healthy diet sustainably produced	• Introduce and enforce mandatory guidelines for provision of healthy food in public institutions (e.g., schools, hospitals, military, prison and other government institutions).	• 2005: The National FBDGs was developed and introduces as one of the main tools to raise awareness regarding healthy diet. Second version of the FBDG was introduced in 2016 (24).	Reevaluating the national FBDG, as well as the thrift food basket in terms of food grouping and recommendations in order to make it more sustainable.
Regulation of marketing of foods and non-alcoholic beverages, including breastmilk substitutes	• Implement the WHO Set of recommendations on marketing of foods and non-alcoholic beverages to children • Reinforce the package of policies and interventions to promote, protect and support breastfeeding and appropriate complementary feeding.	• 1995: Code of marketing of the breast milk substitutes was initiated in the country (72). • The 5th national develoment plan (2011–2016), bans advertising unhealthy goods and services to the public (63). • 2014: Healthy school canteen program was initiated to regulate access to healthy foods in schools (73). • 1978: Food advertisements in school and educational facilities are banned (74).	“Water penalty” and the “water tax” for food products with high water footprint (75). Eliminate legal barriers and disproportionate food-safety standards that lead to high waste rates (71). Investments in water-friendly food processing technologies to tackle natural resources overexploitation and promote corporate profitability (75).
Food products reformulation	• Progressively reduce intakes of salt, sugars and saturated fats by improving the nutritional quality of foods through government-led reformulation programmes. • Eliminate trans fats through the development of legislation to ban the use of industrially-produced trans fats in the food chain	• 2015: Mandatory upper limit of salt in most commonly consumed canned foods e.g., tomato paste and salty snacks, and all types of bread was decreased (1.8%) (59). Also, the standard of salt in cheese was decreased from 4 to 3% and in dough (fermented yogurt drink) was decreased from 1 to 0.8% (59). • 2016–2017: The standard of salt in bread was further decreased to 1% (59). • 2017: The standard of SFA in edible oil decreased to <25 (59). • 2018: Salt use in probiotic yogurts was banned (59). • Food reformulation initiatives for reducing the amount of sugar, salt, and fat, specifically the industrially-produced trans-fatty acids in industrial food products (61, 62).	Decrasing flour milling degree to incease fiber and nutrient content of flour and bread (76). Focus on some of the new technologies being developed to create alternatives to animal products, such as meat, milk and egg, which should reduce the amount of meat consumed. Also, increasing number of fiber-containing products is highly recommended (77, 78).
Front-of-pack labeling	• Implement mandatory standards for ingredient listing, back-of-pack nutrient declarations and simplified front-of-pack labeling for all pre-packaged foods	• 2014: MOHME launched Front-of-Pack nutrition labeling policy (as traffic light scheme) with the objective of reducing sodium, trans fatty acid and sugar intake in accordance with the national action plan for control and prevention of NCDs (63).	Food labels including information about links between food and climate chang are effective in encouraging sustainable food choices (79)
Food fortification	• Implement and monitor policies and practices for wheat flour fortification and for salt iodization, in line with the latest WHO recommendations on best practice.	• Mandatory flour fortification with iron and folic acid was implemented in one province from 2001 and expanded simultaneously to the other provinces in Iran (80). • since 2000, Iran has been recognized by WHO-as an IDD free nation through integration of IDD control into the health network and mandatory iodization of household salt (81). • Voluntary fortification of edible oil, milk, cakes, and pasta with vitamin D (82).	Universal fortification along with small dietary shifts represents an approach to improve the vitamin D status of the general population, at a high acceptability without affecting the carbon footprint (83)

To the best of our knowledge, this study is the first study on current literature on the sustainability of the Iranian diet and recommended changes. The findings can serve to guide policymakers and planners of the food and nutrition food system to develop and implement policies to shift to a sustainable dietary pattern in Iranian population. This study also has several limitations that need to be considered in evaluating its findings. The quality of the studies included in this study was not assessed. Thus, the conclusions are based on the existence of studies rather than their intrinsic quality. Moreover, the Iranian food intake values based on a nutritional assessment were not available for comparison with the recommended values.

## Conclusion

This study showed that there is an urgent need to accelerate the move toward a sustainable diet in Iran. For this purpose, increasing consumption of dairy, fruits, vegetables, cereals, poultry, and legumes and decreasing bread, rice and pasta, red meat, eggs, hydrogenated fats, sugar, and sweets intake are the main changes suggested. Development of an enabling environment to promote moving toward a sustainable diet will require policy and action to: (1) Improve public awareness and interest; (2) Support study to provide evidence and identify possible alternatives; and (3) Plan and implement interventions/programs to promote and facilitate healthy and sustainable diets. In this regard, defining scientific targets to achieve sustainable diet and food production and re-evaluating existing policies related to food and nutrition system are prerequisites to such transformation.

## Author Contributions

NO and AA contribute to the conceptualization. SS and NO contribute to the data collection, reviewing the studies, analyses, and writing—original draft preparation. NO, ZA, and AA contribute to the writing—review and editing. All the authors have read and agreed to the published version of the manuscript.

## Conflict of Interest

The authors declare that the research was conducted in the absence of any commercial or financial relationships that could be construed as a potential conflict of interest.

## Publisher's Note

All claims expressed in this article are solely those of the authors and do not necessarily represent those of their affiliated organizations, or those of the publisher, the editors and the reviewers. Any product that may be evaluated in this article, or claim that may be made by its manufacturer, is not guaranteed or endorsed by the publisher.
